# Computer Aided Screening of Secreted Frizzled-Related Protein 4 (SFRP4): A Potential Control for Diabetes Mellitus

**DOI:** 10.3390/molecules190710129

**Published:** 2014-07-11

**Authors:** Shazia Anwer Bukhari, Waseem Akhtar Shamshari, Mahmood-Ur-Rahman, Muhammad Zia-Ul-Haq, Hawa Z. E. Jaafar

**Affiliations:** 1 Department of Applied Chemistry and Biochemistry, Government College University, Allama Iqbal Road, Faisalabad 38000, Pakistan; E-Mails: bukhari.shazia@yahoo.com (S.A.B.); waseem_shamshari@yahoo.com (W.A.S.); 2 Department of Bioinformatics and Biotechnology, Government College University, Allama Iqbal Road, Faisalabad 38000, Pakistan; E-Mail: mahmood1233@yahoo.com; 3 The Patent Office, Karachi 74400, Pakistan; 4 Department of Crop Science, Faculty of Agriculture, University Putra Malaysia, Serdang 43400, Selangor, Malaysia

**Keywords:** bioscreening, diabetes mellitus, SFRP4, cyclothiazide, clopamide, perindopril

## Abstract

Diabetes mellitus is a life threatening disease and scientists are doing their best to find a cost effective and permanent treatment of this malady. The recent trend is to control the disease by target base inhibiting of enzymes or proteins. Secreted frizzled-related protein 4 (SFRP4) is found to cause five times more risk of diabetes when expressed above average levels. This study was therefore designed to analyze the SFRP4 and to find its potential inhibitors. SFRP4 was analyzed by bio-informatics tools of sequence tool and structure tool. A total of three potential inhibitors of SFRP4 were found, namely cyclothiazide, clopamide and perindopril. These inhibitors showed significant interactions with SFRP4 as compared to other inhibitors as well as control (acetohexamide). The findings suggest the possible treatment of diabetes mellitus type 2 by inhibiting the SFRP4 using the inhibitors cyclothiazide, clopamide and perindopril.

## 1. Introduction

Diabetes mellitus (DM) is a leading cause of morbidity and mortality throughout the world. The number of patients is expected to rise to 366 million in 2030 as compared to 171 million in 2000. The prevalence of disease in patients living in city areas of developing countries suffering from diabetes mellitus will become double in 2030 as compared to year 2000 [1]. Complications of diabetes mellitus are linked with high rates of hospitalization, blindness due to high sugar levels, renal failure and non-traumatic amputation [2]. Due to malfunctioning of the pancreas that metabolizes glucose, blood sugar levels are increased in diabetic patients, *i.e.*, hyperglycemia and the process of converting carbohydrates into energy does not perform its function properly. This leads to polyuria, polydipsia and polyphagia. Chronic hyperglycemia causes damage to the kidneys, nerves, eyes, heart and blood vessels [3]. Diabetes is characterized into two types, *i.e.*, type 1 and type 2. In type 1 diabetes, the body fails to produce insulin, while type 2 diabetes is caused by insulin resistance. It is also due to altered expression of several genes and their products [4,5].

Secreted frizzled related protein 4 (SFRP4) is a member of the SFRP family. SFRPs act as modulators of the Wnt signaling pathway. A large number of diabetes-associated factors are studied in the Wnt signaling pathway [6,7,8,9,10]. SFRP4 acts on Gi/o-coupled receptors as is evident by the loss of inhibitory effect in the presence of pertusis toxin. Moreover, SFRP4 increases the level of unphosphorylated β-catenin and activated TCF/LEF reporter constructs. Taken together, this indicates that SFRP4 activates canonical Wnt signaling. This is in agreement with the fact that TCF/LEF activation causes decreased Ca^2+^ channel expression. Furthermore, the inhibition of exocytosis by SFRP4 was not influenced by co-treatment with canonical or non-canonical Wnt proteins [11,12]. Individuals having increased levels of SFRP4 in the blood are five times more likely to develop diabetes in the coming years. SFRP4 causes reduction in insulin secretion and it is over- expressed in type 2 diabetes mellitus [13] so the inhibition of SFRP4 results in the proper secretion of insulin leading to control of diabetes type 2.

The use of structure-based drug design is a promising technique to discover novel inhibitors/molecules and is becoming a famous practice in modern drug discovery. The drug is an organic small molecule that is used to activate or inhibit the function of different biomolecules like proteins. This in turn results in a therapeutic benefit to the patient. Drug design involves the construction of small molecules that are complementary in shape and charge to the target with which they interact. Drug design frequently relies on computer modeling techniques [14]. This work is carried out to find potential inhibitors of SFRP4 protein to control diabetes mellitus type 2.

## 2. Results and Discussion

### 2.1. Model Evaluation

**Figure 1:**
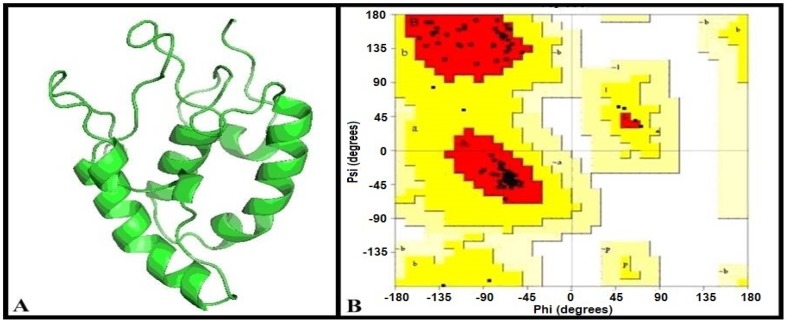
Construction of model for SFRP4 protein and its model evaluation. (**A**): 3D model of SFRP4 protein; (**B**): Model evaluation by Ramachandran plot.

The construction of 3D structure for SFRP4 protein is reported here (Figure 1A). The model was evaluated by Ramachandran plot with the following characteristics: 92.7% core, 7.3 allowed, 0.0% generous, 0.0% disallowed (Figure 1B). The evaluated folding patterns were found similar to the comparative study of various tools for secondary structure prediction. Therefore, the prepared model was good for folding patterns and the RMSD and RMSF values calculated were within the favorable limit of 0.2 nm.

### 2.2. Determination of Active Sites in SFRP4 Protein

**Figure 2:**
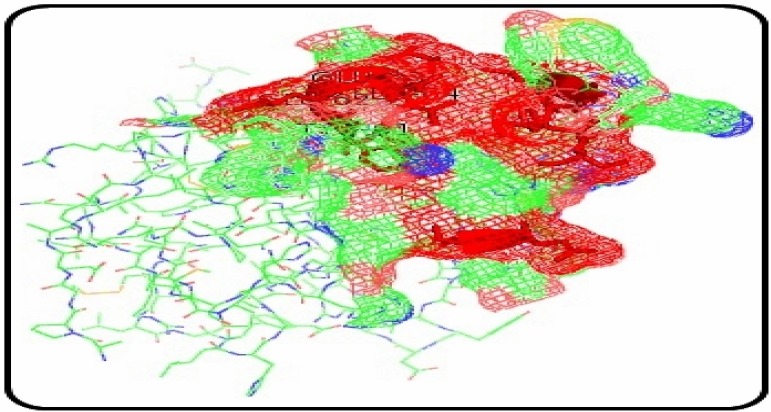
Active sites in the SFRP4 protein.

SFRP4 was found to be folded into two independent domains: the N-terminus containing a secretion signal peptide and a cysteine-rich domain. The active sites of SFRP4 were not clearly mentioned in the literature, so the active sites for binding of ligand were determined by using Pocket finder. According to the results, site 1 contains the amino acids glutamine 60, tyrosine 61, glutamine 63, leucine 64, leucine 111, methionine 112, tyrosine 115, histidine 117, tryptophan 119 (Figure 2). Hence these sites could be inhibited in order to inhibit the function of SFRP4, so that it may not suppress the secretion of insulin.

### 2.3. Interaction Studies of SFRP4 and Potential Inhibitors of Diabetes Mellitus

**Figure 3:**
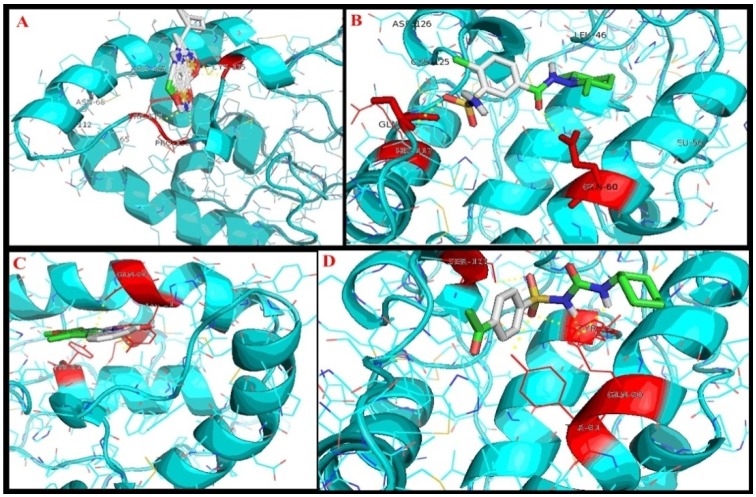
Interaction studies of SFRP4 protein with selected inhibitors. (**A**): cyclothiazide; (**B**): clopamide; (**C**): perindopril; (**D**): acetohexamide (control).

Protein-protein interactions have a very key role in structure-based drug design. We analysed the interactions of some inhibitors and the antidiabetic drug acetohexamide as a control, with insulin suppressor protein Secreted Frizzled-Related Protein 4 and observed that these compounds can inhibit the protein activity by binding to its active site. SFRP4 protein was docked with twelve inhibitors, and three of them were properly bound to the desired site of the protein: cyclothiazide (Figure 3A), clopamide (Figure 3B), and perindopril (Figure 3C) were found to interact with the SFRP4 protein along with the control (acetohexamide; Figure 3D). 

The energy values obtained are shown in Table 1. Energy values are calculated on the basis of arranging the docking results whereby the seed member for drug design is represented by the lowest energy docking solution. The target is predicted by lowest energy orientation [15]. RMSD values were evaluated for the reliable docking results. Nine different poses were prepared by Auto Dock Vina for each ligand and the best pose was selected (Table 1).

The ligand was selected with best dock score and low bound energy. The ligand clopamide pose 2 had affinity (−7.7 kcal/mol) and maximum hydrogen bonds with (His117, Gln60), cyclothiazide with pose 6 had affinity (−8.0 kcal/mol) and maximum hydrogen bonds with (Trp119, Pro120, Gln60) and perindopril with pose had affinity (−7.6 kcal/mol) and maximum hydrogen bonds with (Gln60, Tyr61, Tyr81) as shown in Table 1. Using AUTODOCK VINA depending on the energy values it is conclude that cyclothiazide, clopamide and perindopril are the best and the powerful inhibitors to treat diabetes mellitus.

Thus it can be inferred from the results that diabetes mellitus is a metabolic disorder caused by the deficiency of insulin. In Type 1 diabetes, insulin deficiency occurs due to the destruction of the β-cells of the pancreas. Type 2 diabetes involves decreased insulin secretion or insulin resistance [16]. Insulin is essential for maintaining blood glucose and regulating carbohydrate metabolism. Different inhibitors are used against diabetes. Banana flower flavonoids are used to cure diabetes mellitus as insulin receptor tyrosine kinase activators [17].

**Table 1:** Interaction of SFRP4 protein with some of potential inhibitors. * Residues with best binding affinities.

Ligand Pose	Clopamide		Cyclothiazide		Perindopril		Acetohexamide	
	Affinity (kcal/mol)	Binding Residues	Affinity (kcal/mol)	Binding Residues	Affinity (kcal/mol)	Binding Residues	Affinity (kcal/mol)	Binding Residues
**1**	−7.8	-	−8.5	-	−7.7	-	−7.3	-
**2**	−7.7 *	His117 Gln60	−8.4	-	−7.7	-	−7.2 *	Gln60 Tyr61 Tyr81
**3**	−7.6	-	−8.3	-	−7.6 *	Gln60 Tyr61 Tyr81	−5.9	-
**4**	−7.3	-	−8.1	-	−7.6	-	−5.9	-
**5**	−7.2	-	−8.0 *	Trp119 Pro120 Gln60	−7.5	-	−5.8	-
**6**	−7.2	-	−8.0	-	−7.4	-	−5.8	-
**7**	−7.1	-	−7.7	-	−7.4	-	−5.5	-
**8**	−7.0	-	−7.6	-	−7.3	-	−5.5	-
**9**	−6.9	-	−7.5	-	−7.3	-	−5.5	-

Type 2 diabetes mellitus is caused by decreased insulin secretion. It is reported that people having above normal levels of SFRP4 produce less insulin, which results in decreased carbohydrate metabolism [13]. It has been found that sFRP4 selectively induced apoptotic events in endothelial cells by increasing cellular levels of reactive oxygen species [18]. It has also been demonstrated that SFRP4 interacts with the Wnt signaling pathway and correlated with inflammatory markers. Further studies have indicated that interleukin-1β induced its secretion from islets [19]. Thus, by inhibiting SFRP4 up to a certain level we can increase the level of insulin in human body and reduce the expression of Ca^2+^ channels [13]. Thus it could be inferred from the literature that SFRP4 could be used as a biomarker for detecting type 2 diabetes from the sera of disease persons [20]. Recent studies related to SFRP4 functionality shows that it is a potent tumor-causing phosphaturic agent. SFRP4 is a circulating protein that can be detected several years before the onset of the disease. SFRP4 shows phosphatonin-like properties, because it is a circulating protein that promotes phosphaturia and hypophosphatemia [21].

Thus there is a need to stabilize the expression of SFRP4. This can be done by using different inhibitors that successfully downregulate the expression of SFRP4. Different inhibitors could be used against SFRP4 like cyclothiazide [22]. Acetohexamide is used to treat diabetes mellitus type 2. It causes an increase in the concentration of insulin produced by the pancreas. It also helps body to metabolize insulin more efficiently.

The activated SFRP4 reduces the secretion of insulin if individuals have above average levels of protein and are five times more likely to experience diabetes [13]. The active regions were found enriched with residues Gln60, Tyr61, Glu63, Leu64, Val67, Tyr81, Leu111, Met112, His117, Trp119, Pro120 and Cys125. Twelve different inhibitors were docked with protein SFRP4, and three inhibitors (cyclothiazide, clopamide and perindopril) interacted significantly with SFRP4.

The docking analysis resulted in the detection of important inhibitors that are compatible with the binding site of the targeted protein. As a result of this study we can conclude that the inhibitors computationally studied here have shown good relationships between IC_50_ values, docking score and binding interactions, so these compounds cyclothiazide, clopamide and perindopril may be potent drug candidates and their potency against SFRP4 may be increased.

Along with these finding we should raise our national standards of care for people with DM. People should be educated about the fall-out of this malicious disease by educational programmes and media campaigns since prevention is better than cure in this disease. Routine check-ups by doctors are necessary and the first step to control this disease while specific diets and nutritional cures are the next. Even when this disease is developed, it may be cured if properly treated.

SFRP4 protein sequence (Accession No. CAG46532.1) was retrieved from NCBI database [23]. The protein under study has 346 amino acids. Then potential inhibitors of SFRP4 were retrieved from ChemIDplus [24] and PubChem [25] in SDF format as 2D structures (Table 2), which were then converted into 3D structures by using Open Babel software. 3D structure of SFRP4 protein was modeled by using online server Phyre2 [26] with >90% accuracy and was evaluated by Ramachandran plot.

**Table 2:** List of inhibitors docked with target protein, SFRP4. * Drugs which shows proper interactions with SFRP4.

Sr. No.	Drug Name	Molecular Formula	Drug ID (ChemID)
**1.**	*Captoril*	C_9_H_15_NO_3_S	44093
**2.**	Cyclothiazide *	C_14_H_16_ClN_3_O_4_S_2_	2910
**3.**	*SFRP-1 Inhibitor*	C_19_H_26_N_2_O_4_S_2_	25147677
**4.**	Clopamide *	C_14_H_20_ClN_3_O_3_S	12492
**5.**	*Sulfonamides*	H_2_NO_2_S	12145736
**6.**	Perindopril *	C_19_H_32_N_2_O_5_	107807
**7.**	*Ramipril*	C_23_H_32_N_2_O_5_	5362129
**8.**	*Benazepril*	C_24_H_28_N_2_O_5_	5362124
**9.**	*Lisinopril*	C_21_H_31_N_3_O_5_	5362119
**10.**	*Enalapril*	C_20_H_28_N_2_O_5_	5362032
**11.**	*Acetohexamide*	C15H20N2O4S	1989

The active sites of SFRP4 were found by using Pocket-Finder [27,28]. Pocket Finder is used to detect active sites by ligand binding site detection algorithm Q-SiteFinder. The designed ligand and receptor interactions were performed using AutoDock Vina [29].

## 3. Conclusions

Diabetes mellitus is a life threating disease globally and in Pakistan as well as in rest of world, its prevalence is increasing at an alarming rate. A person suffering from heart disease, lung disorder and hepatic liver infection may also show high levels of blood glucose. Due to this drawback, treating high blood sugar alone is not a guarantee of curing diabetes. The levels of SFRP4 are found to be high in this disease, which results in less insulin production, leading to decreased carbohydrate metabolism. In this study by using the *in-silico* approach we have found some potential inhibitors against SFRP4, which in the future may pave the way for diabetes mellitus cures by decreasing the expression of SFRP4 and increasing insulin production.
